# A feasibility study of expert patient and community mental health team led bipolar psychoeducation groups: implementing an evidence based practice

**DOI:** 10.1186/1471-244X-13-301

**Published:** 2013-11-11

**Authors:** Katharine Coulthard, Dipty Patel, Clare Brizzolara, Richard Morriss, Stuart Watson

**Affiliations:** 1 Newcastle Cognitive and Behavioural Therapy Centre, Carliol Place, Newcastle Upon Tyne NE1 6UR, UK; 2 Druridge Ward, St Georges Park, Morpeth, Northumberland NE61 2NU, UK; 3 Faculty of Applied Sciences, University of Sunderland, Room 105, Dale Building, City Campus, Chester Road, Sunderland SR1 3SD, UK; 4 Institute of Mental Health and CLAHRC NDL, University of Nottingham, Triumph Road, Nottingham NG7 2TU, UK; 5 Wolfson Unit, Campus for Ageing and Vitality, Institute of Neuroscience, Newcastle University, Newcastle NE4 5PL, UK

**Keywords:** Bipolar disorder, Group psychotherapy, Health plan implementation, Health services research, Professional education, Qualitative research, Psychoeducation, Peer specialist, Expert patient, Service user interventions

## Abstract

**Background:**

Group psychoeducation is a cost effective intervention which reduces relapse and improves functioning in bipolar disorder but is rarely implemented. The aim of this study was to identify the acceptability and feasibility of a group psychoeducation programme delivered by community mental health teams (CMHTs) and peer specialist (PS) facilitators. Organisational learning was used to identify and address systematically barriers and enablers, at organisational, health professional and patient levels, to its implementation into a routine service.

**Methods:**

A systematic examination of barriers and enablers to a three day training process informed the delivery of a first treatment group and a similar process informed the delivery of the second treatment group. Triangulation of research methods improved its internal validity: direct observation of training, self-rated surveys of participant experiences, group discussion, and thematically analysed individual participant and facilitator interviews were employed.

**Results:**

Barriers and enablers were identified at organisational, educational, treatment content, facilitator and patient levels. All barriers under the control of the research team were addressed with subsequent improvements in patient knowledge about the condition and about local service. In addition, self-management, agency and altruism were enhanced. Barriers that could not be addressed required senior clinical and education leadership outside the research team’s control. PS and professional facilitators were successfully trained and worked together to deliver groups which were generally reported as being beneficial.

**Conclusion:**

Psychoeducation groups involving CMHT and PS facilitators is acceptable and feasible but their sustainment requires senior leadership within and outside the organisation that control finance and education services.

## Background

Bipolar disorder is one of the largest medical contributors to disability [1]. It is a highly recurrent disorder with a lifetime prevalence of around two percent. Approximately 10 percent of patients will end their life by suicide [2]. Bipolar disorder is associated with interpersonal stress, job related difficulties, high unemployment, high dependence on benefits, low annual income, high work absenteeism, low work productivity, poor overall functioning, lower quality of life, difficulty maintaining long-term relationships and reduced overall social, physical and emotional well-being [3].

Relapse rates are reduced, but not eliminated, in efficacy trials of mood stabilising drugs such as lithium and sodium valproate. However, there is a significant efficacy-effectiveness gap and response is often associated with persistent neurocognitive impairment [4] and psychosocial dysfunction.

Psychoeducation is widely recommended [5,6] and has randomised controlled trial evidence for an increase in time to manic [7] and depressive [8-10] relapse in the short term. In addition Colom’s group [11] showed significant reductions at 5 year follow up in number of illness episodes, days spent unwell, hospitalisations per patient, and days in hospital per hospitalisation. Despite this, psychoeducation is not widely available in routine care, partly because its delivery is not supported by current service models. It therefore appears prudent to seek to implement this evidence based practice [12].

Community mental health teams (CMHTs) are the standard service providing input for patients with secondary level needs in the UK. They are multidisciplinary teams of medical, nursing, social work, occupational therapy and/or clinical psychology staff usually in a community base away from the hospital, which provide assessment and treatment to those with enduring mental health conditions. However the evidence that standard CMHT input helps patients with bipolar disorder is insubstantial [13].

There is an increasing use of peer specialists (PS, defined as individuals with severe mental illness who use their experience to provide services for other people with severe mental illness) in mental health services, particularly in the United States of America [14,15]. It is argued that this development is supported by social learning theory [16] and that PSs can effectively teach coping and self-management strategies [17] and further that PSs often increase patients’ engagement and improve patient satisfaction [14,18]. There is now experience of PSs facilitating groups in mental health settings e.g. [19].

Our model proposes a manualised psychoeducation group delivered by CMHT workers and PSs working together under expert supervision within the CMHT. These groups should be financially more viable than groups provided by a specialist mood disorder service [11], or psychologists. Moreover, the groups should help enhance the skillset of CMHT workers and should allow the intervention to be delivered by the service which will also be providing other aspects of care including crisis response.

This paper outlines a feasibility study primarily run to assess the acceptability of the proposed group psychoeducation programme and to identify drivers and barriers that may impact on the rolling out of the programme. It outlines how these were identified and addressed, and examines whether groups can be successfully run in this format.

## Method

The pilot research was conducted in accordance with the Helsinki Declaration and prior approval of the Local Research Ethics Committee was obtained.

### Groups

The proposed group psychoeducation programme was a series of 14 weekly sessions. The groups were structured and involved a review of the previous session and homework with some didactic teaching, interactive small group and individual exercises followed by homework assignments. There was a weekly hand-out describing the session which was used by facilitators and participants and was designed to be kept as a reference source. The first seven sessions were based on the early warning symptoms intervention [7] and were aimed at teaching group participants to recognise their own individual relapse signature and to take appropriate action in the early stages of relapse. In addition, there were three sessions on medication, and three sessions on lifestyle issues including sleep routines and use of alcohol and drugs. The final session was devoted to review and feedback. Individual attendance by participants and facilitators was recorded.

### Organisational structure

The groups were part of a new bipolar disorder service which used a hub and spoke model. The hub included a nurse specialist with consultant psychiatrist (SW) support. The CMHTs formed the spokes. As such it was envisioned that the bipolar service would develop as an expert resource to provide direction, training, administrative support and evaluation of and support for the groups. The groups themselves would be run by CMHT staff in conjunction with PS facilitators, and overall responsibility for patient care would remain with the CMHT.

Key to this strategy was the appointment of a full time nurse specialist (DP) in the new bipolar service who would take a lead role in all aspects of the groups’ organisation. The bipolar disorder service was funded initially with two grants from the pharmaceutical industry. A steering group was set up within our health care organisation to present a business case for the groups and the new service. Funding was later secured for the clinical nurse specialist position via the health care organisation on a short term rolling contract.

### Facilitator training and support

Potential health professional and PS facilitators were identified through communication with CMHTs. Potential facilitators attended a joint training programme (for professionals and PSs). This consisted of an initial three-day workshop conceived as an introduction to facilitating groups and to the key concepts of the programme followed by 12 further weekly training sessions.

The training was supported by, and run in conjunction with, Bipolar UK, a UK based national charity which promotes self-help opportunities for people with bipolar disorder and has a history of running successful group based self-help courses.

It was planned that facilitators would have weekly supervision from a trained group therapist commencing three weeks before the first group psychoeducation session and continuing until one week after the last session. It was also planned that facilitators would receive practical support during the groups which would be provided by the nurse specialist.

### Data collection and analysis

**Figure 1:**
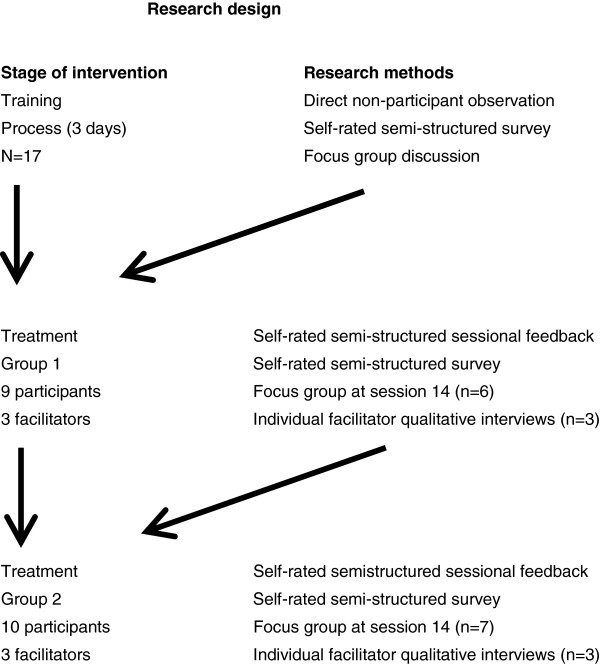
Research design.

An organisation learning approach was taken to the data collection and analysis [12]. It is both a way of measuring barriers and enablers to change and a model of implementation based on that information. This approach to implementation was developed to enable business to adapt swiftly to constantly changing market conditions. There are many parallels between the business world facing constant change and the constant change in response to social, political, financial, technological context in the National Health Service in England [20] and other parts of the world. There was a systematic examination of barriers and enablers to the intervention; these were considered from organisational, educational, therapy delivery, health professional and service user perspectives. The iterative nature of knowledge mobilisation was recognised [12], hence data at each stage of the intervention from training to group 1 and then from group 1 to group 2 was used to improve implementation by informing the development and delivery of the next stage of the intervention. The design of the study is shown in Figure 1.

In this study, inductive research methods were required to produce valid findings relatively efficiently and quickly so they would be available for use to inform the next stage of the intervention. To increase the internal validity of the findings we used methodological and data source triangulation [21,22] for the training process and each of the treatment groups. Triangulation refers to the process of enhancing internal validity and providing more detailed and multi-layered information by using multiple methodological sources to gather data to determine if there is a convergence [23]. At each stage we used two methods to generate inductive data and used a third (focus group discussion or individual qualitative interviews) to explore consensus, complementarity and dissonance in data set comparability, and to collect further data and make further comparisons in terms of defining barriers and drivers to the intervention [12,22,24,25].

The methods of data collection in the training process were: i) direct non-participant observation of the three day training process by KC who collected field notes and analysed these thematically after the training [26]; ii) self- completed semi-structured questionnaires using Likert scales e.g. “Overall I found meeting health professionals at the training a positive experience” with room after each question for open responses [25,27] (see Table 1). These were collected on completion of the overall training process; iii) individual and group discussion involving all participants in the training process led by KC to explore the experience of the training process by participants and also, the barriers and drivers to the training [28]. This was conducted on completion of the overall training process. Specific qualitative data software was not used.

**Table 1:** Results of semi-structured questionnaires from facilitator training

**Question (1 = disagree, 9 = yes definitely)**	**n**	**mean**
Overall I found attending the facilitator training a positive experience	8	8.1
Overall I found meeting people with bipolar disorder during the training a positive experience	8	7.8
Overall I found meeting health professionals at the training a positive experience	8	7.1
By attending the facilitator training I learnt more about bipolar disorder	8	7.8
By attending the facilitator training I learnt more about ways of managing bipolar disorder	8	8
The way I work with people with bipolar disorder has improved as a result of doing the training	8	6.8
My mental health has improved as a result of the training	8	4.2
My mental health has worsened as a result of the training	8	1.7
I was satisfied with the room and the facilities where the training occurred	8	5.6
I made useful contacts through attending the training	8	6.4
After the training I felt I was ready to become a facilitator	7	5.8
After the training I decided I would like to become a facilitator at some time in the future	7	6.1

A different approach was taken to the analysis of barriers and drivers to the intervention in the treatment group as direct participant observation might have been inhibiting to the group psychoeducation process. Semi-structured questionnaires delivered weekly after each treatment session were designed by KC on the basis of data obtained from the analysis of barriers and drivers in the training process to elicit participants’ opinions of the content and structure of individual sessions. As in the training process questionnaire, items were answered using Likert scales with room after each question for open responses. A final questionnaire on overall satisfaction with the groups and achievement of goals from the treatment groups was also completed at the end of the last treatment group session (see Table 2). Each questionnaire was completed individually and independently by the participant. KC met with the participants as a group at session 14 to elicit their experiences of the group. These discussions were analysed thematically by KC [29] and each code was discussed with the consultant psychiatrist before final coding as a barrier or enabler to the intervention (Table 3).

**Table 2:** Satisfaction and goals questionnaires from psychoeducation groups

**Statement (1 = strongly disagree, 9 = strongly agree)**	**Treatment group 1**	**Treatment group 2**
	**Mean (s.d.)**	**Mean (s.d.)**
	**N = 5**	**N = 7**
Are you satisfied with the programme overall?	9.0 (0)	9.0 (0)
Did you find meeting other people with bipolar disorder a valuable experience?	9.0 (0)	9.0 (0)
Was an expert patient as a facilitator helpful to the overall experience of being in the group?	8.8 (0.5)	8.6 (0.8)
Do you feel that the symptoms of your illness have improved as a result of coming to the group?	6.0 (1)	8 (1.9)
Did attending the psycho-education group help you understand your illness?	8.4 (0.9)	8.6 (0.8)
Did the groups help you find new ways to cope with having bipolar disorder?	8.4 (0.9)	8.3 (1.1)
I understand what a relapse signature is	9.0 (0)	7.9 (1.6)
I am aware of triggers for my illness	8.6 (0.9)	7.8 (1.5)
I know what my warning symptoms are for depression	8.0 (1.4)	7.7 (2.0)
I know what my warning symptoms are for mania	9.0 (0)	8.4 (1.1)
I am aware of coping strategies to use at different stages of my illness	8 (1)	7.6 (1.81
I use different coping strategies at different stages of my illness	8 (0.7)	7 (2)

Results shown from group members who completed the group and questionnaires.

**Table 3:** Summated feedback from weekly post psychoeducation group semi-structured questionnaires

	**Number of responses**	**Mean response**
Item 1. How interesting did you find the session? (1 = not 10 = very)	69	9.1
Item 2.How easy or difficult did you find the material? (1 = too easy, 10 = too difficult, 5 just right)	69	5.6
Item 3. How useful was the session? (1 = not, 10 = very)	69	8.9
Item 4. How much material was covered in the session? (1 = too little 10 too much, 5 just right)	61	5.4
Item 5. Did you feel able to ask questions if you didn’t understand the material? (1 = not at all, 10 = definitely)	62	9.4

After each group session, participants were asked to complete a questionnaire; the responses have been summated and presented above. The questionnaire included a free text option and the responses have been analysed. The following themes emerged.

(1) **Group Processes**. There were comments about the generic benefits of working in a group as illustrated by the following: 'I enjoyed talking about my experience’, 'exchanging ideas’, 'group work’, 'team work’, 'talking as a group’, 'hearing others experiences’, 'group discussion’, 'debate and conversation’.

(2) There were also positive comments about the **learning tools** used e.g. flip charts, feedback, card sorting.

(3) **Specifics of the Session**. There were positive comments about the provision of information and the discussion on insight (5), coping strategies (4) and action plans (2). There were also positive comments about discussions linking high and low symptoms, different forms the illness takes in different people, medication topics (3), support networks, discussion on bipolar I and II and hospitalisation.

(4) **General comments.** Participants reported that the groups were “well presented”, “interesting”, “informative” and said that “we could have had more time”.

## Results

19 trainee facilitators attended the initial three day training. There developed a core group of 8–10 trainee facilitators who then attended the twelve weekly sessions that followed. Eight trainee facilitators filled in the semi-structured questionnaires at the completion of the training. Eight trainee facilitators views were gained via discussion, regarding the training process.

### Issues identified during joint PS and professional training to prepare for the role of facilitators

The initial three day training in 2004 for both trainee PSs and professional facilitators was set up as the first stage towards becoming a facilitator. We had anticipated that this and twelve weekly sessions would be sufficient to then allow facilitators to begin their role. However during the three day training it became apparent that knowledge of bipolar disorder in specific formal areas in trainee PSs was low. For example, despite having long clinical histories, three trainee PSs only reported finding out their diagnosis shortly before attending the group, one as part of the process of being referred to be a facilitator. Confidence among trainee PSs varied greatly from person to person. Some patients found the training process too much, and found it difficult to participate. One trainee PS commented on the burden of meeting others with bipolar disorder *'I felt totally stressed out because of the others, not the training’*. Others were keen to start the role, when it was felt (by S.W. or K.C.) that they had significant further training needs. For example some people were still in the midst of personal experience, either due to illness or other factors that limited their ability to be reflective or to encourage reflection in others. For most trainee PSs it was felt that rather than progressing directly to the facilitator role it would be more appropriate if the next stage in training were to be a participant in the group psychoeducation programme.

Large and medium sized structured exercises which focused on sharing personal experiences of bipolar disorder worked well in that trainee PSs were vocal, and seemed empowered to take the expert role in sharing their experiences. There was colourful use of language and sharing of individual examples which illustrated important concepts being discussed, such as early warning signs, or coping strategies. In small group exercises, of twos and threes, there seemed to be less spontaneous participation. Health professionals and trainee PSs appeared to draw back into traditional roles of advice giving and seeking respectively and health professionals appeared to take the lead in structuring the exercises.

There were major issues around expense reclamation. There were concerns that disclosure of national insurance numbers on health care organisation expense reclamation forms had the potential to jeopardise social security benefit payments. There were significant delays and difficulties in reimbursement through the health care organisation.

A number of changes were made to the proposed content and structure of the programme as a result of feedback from the training days. Most importantly the proposed facilitator structure was changed from two to three facilitators This was identified by trainee PSs as important, as it gave the confidence to know that illness or relapse wouldn’t jeopardise the groups. It also had the potential to be a useful training structure so that a new PSs could work alongside more experienced colleagues in an apprenticeship model. We did not make any changes due to the apparent issue of patients feeling inhibited in small group discussions with health professionals. We believed that this barrier was less likely to recur in the treatment groups because there would be a higher ratio of patients to health professionals, and therefore group exercises would more closely resemble those that had worked well in training.

An example of content being changed was the 'life chart’ exercise [30] being removed, as trainee PSs felt it was likely to be too emotive within a group setting. They felt it would be more appropriate for individual follow up sessions.

We have summarised the enablers and barriers in the training process from data elicited through participant observation, questionnaire and focus groups and the actions that were taken before treatment group one started are shown in Table 4.

**Table 4:** Drivers, barriers and actions to address barriers to delivery of group psychoeducation in the training process

	**Enabler**	**Barrier**	**Action to address barrier**
Organisation outside group		Expense reclamation for patients	Advocacy for patients with health care organisation
Organisation within groups		Small mixed groups of patient and health professional inhibited discussion	Higher numbers of participants to facilitators in groups
Education of participants		Low level of knowledge about bipolar disorder in some patients	Patient becomes a participant in group before becoming a facilitator
Content of treatment		Life chart too personal and emotive	Remove life chart from content of groups- advise for individual follow up work.
Health Professional	Willingness to work with patient	Take lead too much in small groups	Higher numbers of participants to facilitators in groups
Patient	Willingness to work with health professional	Burden of training and low confidence of some patients	Increase number of facilitators from two to three and use apprentice facilitator with more experienced facilitators.

### Treatment group 1

The first group ran from April to August 2005. The facilitators were a community psychiatric nurse (CPN), an inpatient nurse from the affective disorder service and a PS.

### Group participants

Patients diagnosed with bipolar I or II disorder were identified by mental health professionals within local community teams and invited to pre-assessment, where a structured diagnostic interview [31] and clinical ratings [32,33] were performed. The groups were designed to be as inclusive and naturalistic as possible. Exclusion factors were those which might significantly impair learning within a group setting, so consisted of a current DSM-IV alcohol or drug dependence or a current or recent (within the last 3 months) episode of hypomania, mania, mixed mood state or severe depression.

There were nine participants in treatment group 1, they had a mean age of 44 (range 30–60) and a mean age of onset of 27 (range 19–46). Eight participants were diagnosed with bipolar I and one with bipolar II. The mean number of previous hospitalisations was 3 (range 0–10). Seven participants were in remission (mean Hamilton depression rating scale (HDRS) score = 3.4, SD = 2.9), and two were moderately depressed (HDRS = 14 and 15) at the pre-assessment.

### Identification of issues with facilitators post treatment group 1

Unfortunately we underestimated the length of time that it would take for the nurse specialist position to be approved and filled, with the result that the organisational support for the first group was compromised.

The lack of the nurse specialist at the beginning of the groups was important. Facilitators did not feel well supported by the bipolar service and felt burdened with administrative tasks such as photocopying. The nurse specialist’s arrival half way through the first treatment group was experienced by some facilitators as disruptive.

Although facilitators felt well supported in group supervision, the confidentiality of supervision was maintained. This meant that important issues were not fed back to the bipolar service until the end of the group.

One of the professional facilitators, a CPN, discontinued after the sixth session. The normal CMHT case load had not been reduced. *'I discontinued due to pressure of work, which was not reduced therefore 120% work load was being managed, my physical health deteriorated and I had to pull out as a facilitator’.* This left two facilitators to manage the remainder of the sessions.

The PS facilitator discontinued after the thirteenth session. Issues identified were perceived pressure to continue when not feeling well, perceived lack of support, and feeling 'in the middle’ between patients and facilitators. Workload issues were likely to have been accentuated by the departure of the professional facilitator.

'When I began to feel ill and finding it difficult to cope, I hid this as much as I could because of my personal pride. I didn’t want to worry the other facilitators/organisers. I didn’t want to fail or let people down, but in a totally irrational way I felt people should have noticed I wasn’t well, and the fact they didn’t meant they didn’t care, I was just a resource to use’.

'As the only expert patient on the course I didn’t fit easily in either group, facilitators or patients,- I had loyalties to both but was firmly placed in the middle which was a very lonely place a times’.

'Each expert patient is just that- a patient, and each person who is bipolar experiences it in their own unique way. The problems I had may not be a problem at all to other expert patients. My only suggestion is that greater monitoring of the expert patient is vital to their health and the success of the groups’.

### Experiences of working with a mixture of PS and health professional facilitators

This was largely very positively reported by facilitators as illustrated by the following quotes.

'The presence of the expert patient seemed to smooth some of the anxiety normally felt at the beginning of a formal course run by professionals’;

'Working with another professional and a service user was excellent. If all service users acting as facilitators are as good as the one we had, the groups will work well. She brought clarity, humour and dedication to the group. We learnt from each other and the group’.

'I learnt more about ways of managing bipolar disorder particularly from people with the illness, and their own strategies’.

### Treatment group 2

The second group ran from April to July 2006. The facilitators were the nurse specialist, a CPN and a PS. The second group started with ten participants and seven completed, those seven participants had a mean age of 36 years (range 29–47) and a mean age of onset of19 years (range 7–33). Of these seven, five were diagnosed with bipolar I and two with bipolar II. The mean number of previous hospitalisations was 1 (range 0–3). Six of the completers were in remission (mean MADRS [34] = 7; standard deviation (SD) = 5.7) and one was moderately depressed (MADRS = 21) at the pre-assessment. The three participants who dropped out had a mean MADRS of 10; SD = 6).

Table 5 illustrates barriers and drivers to the delivery of group psychoeducation in treatment group one elicited from weekly and final questionnaires and group interviews, and changes made for treatment group two. A number of changes were put into place for treatment group 2 regarding facilitator support to overcome the problems with the facilitator role that were experienced in treatment group 1. Fundamentally the nurse specialist was employed before the group started. There was a resultant improvement in organisation of the group and support for the facilitators. There were planned meetings for the facilitators with the bipolar disorder service to ensure earlier communication if facilitators were experiencing difficulties. Regular supervision with the group therapist continued, but with clear rules between facilitators, the supervisor and bipolar disorder service about confidentiality and information sharing. All facilitators met with KC or the consultant psychiatrist prior to the group to discuss advance agreements, potential difficulties and avenues of support. A document regarding pathways of support was distributed to facilitators. The PS from the first treatment group offered to act as a mentor to the PS for group 2.

**Table 5:** Drivers, barriers and actions to address barriers to delivery of group psychoeducation from treatment group 1 to treatment group 2

	**Enabler**	**Barrier**	**Action to address barrier**
**Organisation outside group**		Health professional not released from other duties in post.	Negotiated temporary reduction in other duties while facilitating group.
		Delay in appointment of bipolar disorder nurse specialist.	Groups run when bipolar disorder nurse specialist in post.
**Organisation within groups**		Some important issues in supervision not divulged over concerns about confidentiality of supervision.	Discussion and reaching of consensus about which information and issues that are discussed in supervision can be shared with bipolar service.
**Health Professional**	Willingness and positive experience of working with patient facilitator	Need to provide administrative support to facilitator role	Dedicated bipolar disorder nurse specialist planned and undertook administrative tasks instead of health professional
**Patient facilitator**	Willingness and positive experience of working with health professional	Lack of support, especially after health professional left and feeling unwell.	Improvements in communication of important issues raised in supervision to bipolar service. Advance agreements about discussions about arrangements if unwell introduced before group starts. Release of health professionals from other duties. Mentoring offered from former expert patient facilitator
Participants	High acceptability levels, good retention of participants through the programme		

There was negotiation and agreement between the nurse specialist and CMHT managers on reduction of case loads, and clear communication about the time required for groups, preparation and supervision for the health professional facilitators.

### Results of changes made for group two

Facilitators were very positive about their experience of facilitation. All facilitators finished the groups with no reports of ill health as a result. Both health professionals were keen to continue, the PS was undecided. The CPN experienced difficulties attending all group commitments due to pressure of acute CMHT work, despite initial management agreement about attendance.

### Group acceptability

In treatment group one, nine patients started the first group, and six (67%) completed. Drop outs were after sessions one, three and nine. In treatment group 2, ten patients attended at least one group session, and seven (70%) completed the group. Unfortunately we were unable to ascertain the reasons for group drop outs.

The following quotation is illustrative of the gaps in standard care that people had experienced.

'Most of the group were unaware that they were diagnosed bipolar until they were asked to participate in the project’. 'Almost without exception the group had never met/spoken to another person who was bipolar’.

Quantitative measures (Table 2) indicated high levels of satisfaction with the group, and high self-reported attainment of goals in those who completed the questionnaires. Patients reported that the group had helped them to understand their illness, feel more in control of it and to find new ways of coping with it. They had valued the experience of meeting others with bipolar disorder.

Analysis of the group discussions and semi-structured questionnaires supported the quantitative results. Participants reported finding the group extremely useful, and liked the structure and content.

Table 6 highlights the major drivers identified by participants and facilitators, as well as remaining barriers.

**Table 6:** Remaining drivers, barriers and actions required to embed delivery of group psychoeducation in routine mental health care

	**Enabler**	**Barrier**	**Action to address barrier**
Organisation outside group		Lack of senior clinical leadership to support funding of intervention and bipolar disorder nurse specialist	Reappraisal of costs and benefits of intervention in light of research evidence and competing demands for resources
		Insufficient detailed training of crisis resolution and home treatment team, community mental health team, accident and emergency and primary care in early warning sign interventions in bipolar disorder	Investment in training and senior educational and clinical leadership to support such training
Organisation within groups		Lack of understanding of reasons for drop outs from groups.	Research directed at understanding and addressing reasons for drop out from groups.
Facilitators	Willingness and positive experience of health care professional and patient facilitators working together	Training structure relatively fixed. Insufficient reward and ongoing support for facilitators.	Create a sustainable, and flexible structure for training. Find ways to reward and provide ongoing support for facilitators. Embedding training and support systems within local clinical and education service provision.
Participant	Shared information giving about local resources	Informal support and psychoeducation largely independent and unknown to local mental health services.	Engage services with working positively with third sector and service user organisations to embrace recovery principles of care.
	Commonalities in dealing with illness		
	Newly diagnosed learning from older participants		
	Knowing more about illness		
	Improvements in agency		
	Altruism to help others		

In the second group, spontaneous reports of significant life changes following the group were impressive. These included specific reports of people taking actions believed to have averted relapse. Five members volunteered for further service user work with a local user and carer network (UCAN) and two applied to join the 'Service user research group in England’ (SURGE). One group member returned to full time education, and another to paid work, including employers in his action plans for relapse. Overall the treatment groups resulted in improvements in agency i.e. a greater willingness and confidence to take control of their illness with a view to preventing relapse or reduce its overall impact. Group participants commenced negotiations with the local bipolar UK group to set up a new branch, with more convenient evening meetings. They became heavily involved in further promotion of the psychoeducation groups, visiting and giving advice to other areas planning to set them up.

The barrier that in this case led to the groups not being implemented was that senior management support was not secured for the long-term funding for the nurse specialist. At that time all nursing jobs at that level of seniority were reviewed across the health care organisation and many, such as this, were no longer funded as part of cost savings.

Another important barrier identified was the lack of responsiveness by health professionals outside the CMHT to a group member’s action plan during a crisis presentation. The work of educating services particularly primary care, accident and emergency and psychiatric crisis resolution and home treatment teams about the groups and the 'action plans’ coming out of them remains an important barrier to overcome.

## Discussion

The study provides evidence that the delivery of psychoeducation groups using community mental health team staff and PSs is acceptable to participants and facilitators if they are well supported. It illustrates the required organisational steps required to successfully run such groups. It also illustrates the value of using a variety of qualitative and quantitative research methods to systematically elicit barriers and drivers to implementation within an organisation learning framework so that lessons learnt can be readily applied to improve the implementation of the intervention.

There are a number of methodological limitations to the approach we took. The first is consideration of the method of knowledge mobilisation and implementation research. There are many approaches to both and many of these approaches are reviewed by Rowley and colleagues [12] and Ferlie and colleaguesl [35]. While there is a more settled literature on implementation of evidence into clinical practice in general, there is much less agreement on the best way to do this in specific health care organisations, as we describe here. Therefore, investigators have been invited to consider the theoretical and method approach that is most suitable to the question that is being studied [35]. Organisational learning is an approach that helps business to thrive in changing market conditions but it has rarely been evaluated in health care research and so its validity and utility in health service practice is yet to be established [12]. It values competency in delivering interventions and takes the perspective that a systematic understanding of an organisation’s cognitive capacity at each level of the organisation and as a whole is critical to the delivery of such interventions [35]. To be effective in organisational learning, research methods have to be analysed in time to inform further refinement of interventions while there is still enough momentum and leadership within the health care organisation to deliver them. The data and changes to address the barriers to the delivery of the intervention are an iterative process. The emphasis is on understanding the sociological context of the changes, especially absorptive capacity or the ability to utilise new knowledge at that time at different levels of an organisation [36]. The iterative nature of the implementation approach shares many similarities with participatory action research or continuous quality improvement [37]. Compared with longer term systematic plans of implementation based on a readiness for change model e.g. [38] or other projects used to implement service change in serious mental illness e.g. [15] it is more reactive and concerned with what can be done now. The systematic collection of data to consider barriers at organisational levels and user experience makes this a broader and more dynamic process than could be achieved merely through professional reflective practice.

Potentially there are many limitations of the approach. There may be insufficient time and resource to adequately explore discrepant data that might be important to fully understand barriers to the delivery of an intervention. For instance, in this study we were unable to interview participants who dropped out of the groups who may have been able to pinpoint further barriers to the intervention and probably did not have the same satisfaction with the intervention. We did not interview other clinicians and administrators involved in the care of the patients in these groups or who might refer patients to such groups due to the limitations of the pace of the work and resource to do it. The analysis was also performed by researchers from only one professional background (psychiatry) and might have been enhanced by consideration of the data from a variety of different professionals and service users.

To improve the internal validity of the research methods, we utilised triangulation of research methods and data sources (direct observation, participant perspectives, facilitator perspectives) [21]. However, there is no common agreement about the optimal way to use this technique [22]. There can be incompatibilities between the units of analysis and the research paradigms that might contradict each other or even amplify sources of error and bias [25,39]. In this instance, the methods can all be seen as inductive and focussed on eliciting barriers and enablers to a specific intervention so the most likely error if present at all is to underestimate or to fail to identify some of the barriers to the intervention. The organisational learning process as described here seems to have been effective in addressing the problems that were within the control of the research team. However the barriers to the delivery of the intervention within the health care organisation itself were not addressed sufficiently as none of the research team could provide the necessary senior clinical leadership within the health care organisation that hosted the study to address these barriers [40]. Furthermore there were barriers to the use of the crisis plans developed by participants in the groups that were not enacted upon by primary care, accident and emergency and crisis resolution and home treatment teams. The lack of knowledge concerning how to utilise these plans might be addressed through education. This would require leadership and planning outside the mental health care organisation as well as within; an argument for a more planned approach to implementation [15].

These groups represent a different model, allowing health professionals and service users to train and work together. The results were particularly positive with regard to participant acceptability. There were high satisfaction ratings, good self-reported goal attainment, and good participant retention. As we had hoped PSs and health professionals did work well together within the treatment groups to create an excellent environment in which patient learning was optimised. This project set out to be collaboration between patients and professionals and this may explain, in part, the high level of acceptability. The experiences of the PSs in this programme mirror the experiences of PSs in other programmes [41]. Ideally there would also be further work on making training more flexible for PSs, developing clear guidelines on readiness to start training to facilitate, and finding flexible ways to reward PSs.

The major issue for on-going implementation for this programme was the inability of the project team to gain enough senior management support to secure longer term funding for key posts. The inability to provide a stable long-term framework for the groups, felt particularly unsatisfactory, as we became increasingly aware of the significant personal investment many PSs were offering. Although there is some evidence that savings in in-patient and other emergency care more than paid for the intervention over a five year period [42], there are undoubtedly initial costs to the programme, in this case nurse specialist and some consultant psychiatrist time. In order for the programme to be sustainable this initial cost would also need to include “backfill” time for the professional facilitators. Importantly, this study took place in the context of a British national health service which was, and is, trying to implement cost savings.

In terms of limitations this pilot has not examined efficacy, but aspects of the intervention’s efficacy have already been demonstrated [7-11]. This study has informed the design of another group psychoeducation intervention involving health professional and expert patients by one of the authors (RM) [43]. However, the new study is not being performed in the same health care organisation as the current study and is not involving local community mental health team staff in delivering the intervention. As a result compared to the current study, the implementation of the intervention into routine clinical practice might be faced with an additional barrier to overcome, namely its acceptance into the service delivered by local community health team.

We conducted this pilot in the UK and have considered it from a UK perspective but recognise that the advantages of making psychoeducation widely available are not confined to our shores and that the ability to deliver such an evidence based intervention to patients with bipolar disorder by training and developing peer specialists and by creating a supportive organisational structure is a global issue.

## Conclusions

Our experience suggests that it is feasible to run a group psychoeducation programme for bipolar patients, with health professionals and PSs working together as facilitators to provide high levels of acceptability for group members. Patients working as facilitators and shaping the intervention may have been important in achieving these high satisfaction levels. The intervention was not implemented into routine practice. However, the feasibility study has allowed us to highlight barriers that would need to be overcome to allow the successful expansion of this programme. These included: maintaining structures for facilitator support; finding ways to allow patients to work sustainably within NHS structures; training staff involved in the delivery of crisis care and on-going support to utilise the early warning signs intervention; obtaining advocacy and support from senior clinical or managerial leaders in the health care organisation to secure long-term funding for a bipolar disorder nurse specialist; and the release from other duties for other health care facilitators.

## Abbreviations

CMHT: Community mental health team; NHS: National health service; NICE: National institute of clinical excellence; PS: Peer specialist.

## Competing interests

The authors declare that they have no competing interests.

## Authors’ contributions

SW was the lead clinician in designing and managing the study, with assistance from KC. RM provided mentorship throughout. CB and DP were fundamental in the delivery of the organisation and delivery of the groups. KC initially drafted the manuscript, with revisions from SW and a major stylistic revision by RM. All authors read and approved the final manuscript.

## Authors’ information

KC is a senior psychiatry trainee, DP held the position of bipolar nurse specialist and is now ward manager on an inpatient unit. CB was a community psychiatric nurse but is now Senior Lecturer. SW is a Senior Lecturer and honorary consultant psychiatrist. RM is the Principal Investigator for PARADES Group psychoeducation versus Group Support RCT, Chair of the NICE Guideline Development Group for Bipolar Disorder (revision) and Director of Research for NIHR CLAHRC NDL which is researching organisational learning as a method of improving implementation. During the writing up of the project, RM was Director of Research for the National Institute of Health Research (NIHR) funded Collaboration for Leadership in Applied Health Research and Care for Nottinghamshire, Derbyshire and Lincolnshire (CLAHRC-NDL), funded by the National Institute for Health Research Collaboration for Leadership in Applied Health Research and Care for Nottinghamshire, Derbyshire and Lincolnshire (NIHR CLAHRC NDL). The views expressed in this manuscript are those of the authors and not necessarily those of the NHS, the NIHR or the Department of Health.

## Pre-publication history

The pre-publication history for this paper can be accessed here:

http://www.biomedcentral.com/1471-244X/13/301/prepub
